# Comparative analysis of EPA and DHA in fish oil nutritional capsules by GC-MS

**DOI:** 10.1186/1476-511X-13-190

**Published:** 2014-12-13

**Authors:** Tao Yi, Shuk-Man Li, Jia-Yi Fan, Lan-Lan Fan, Zhi-Feng Zhang, Pei Luo, Xiao-Jun Zhang, Jian-Gang Wang, Lin Zhu, Zhong-Zhen Zhao, Hu-Biao Chen

**Affiliations:** School of Chinese Medicine, Hong Kong Baptist University, Hong Kong Special Administrative Region, Hong Kong, China; Guangxi Botanical Garden of Medicinal Plant, Nanning, Guangxi 530023 China; The State Key Laboratory of Quality Research in Chinese Medicine, Macau University of Science and Technology, Macau, China; School of Chinese Medicine, Guangzhou University of Traditional Chinese Medicine, Guangzhou, China

**Keywords:** Fish oil, EPA, DHA, GC-MS, Comparative analysis

## Abstract

**Background:**

Fish oil is a popular nutritional product consumed in Hong Kong. Eicosapentaenoic acid (EPA) and docosahexaenoic acid (DHA) are the two main bioactive components responsible for the health benefits of fish oil. Market survey in Hong Kong demonstrated that various fish oil capsules with different origins and prices are sold simultaneously. However, these capsules are labelled with same ingredient levels, namely EPA 180 mg/g and DHA 120 mg/g. This situation makes the consumers very confused. To evaluate the quality of various fish oil capsules, a comparative analysis of the contents of EPA and DHA in fish oil is crucial.

**Methods:**

A gas chromatography–mass spectrometry (GC-MS) method was developed for identification and determination of EPA and DHA in fish oil capsules. A comprehensive validation of the developed method was conducted. Ten batches of fish oil capsules samples purchased from drugstores of Hong Kong were analyzed by using the developed method.

**Results:**

The present method presented good sensitivity, precision and accuracy. The limits of detection (LOD) for EPA and DHA were 0.08 ng and 0.21 ng, respectively. The relative standard deviation (RSD) values of EPA and DHA for repeatability tests were both less than 1.05%; and the recovery for accuracy test of EPA and DHA were 100.50% and 103.83%, respectively. In ten fish oil samples, the contents of EPA ranged from 39.52 mg/g to 509.16 mg/g, and the contents of DHA ranged from 35.14 mg/g to 645.70 mg/g.

**Conclusion:**

The present method is suitable for the quantitative analysis of EPA and DHA in fish oil capsules. There is a significant variation in the contents of the quantified components in fish oil samples, and there is not a linear relationship between price and contents of EPA and DHA. Strict supervision of the labelling of the fish oil capsules is urgently needed.

**Electronic supplementary material:**

The online version of this article (doi:10.1186/1476-511X-13-190) contains supplementary material, which is available to authorized users.

## Background

**Figure 1:**
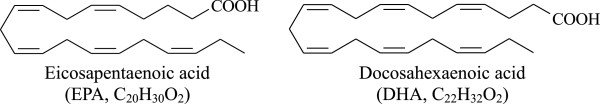
**The chemical structures of EPA and DHA.**

Omega-3 polyunsaturated fatty acids, which include the fish oil components eicosapentaenoic acid (EPA) and docosahexaenoic acid (DHA, Figure 1), are essential for humans as cannot be synthesized by the human body [1]. It has been reported that omega-3 fatty acids are very important in preventing and managing heart disease [2]. Findings show omega-3 fish oil may help to lower blood pressure [3], reduce triglycerides accumulation [4], slow the development of plaque in the arteries [5], reduce the chance of abnormal heart rhythm [6], reduce the likelihood of heart attack and stroke, and lessen the chance of sudden cardiac death in people with heart disease [7]. Omega-3 parenteral nutrition can reduce the rate of inflammatory complications after surgery [1]. It is also thought that EPA in particular may possess some beneficial therapeutic potential in mental conditions, such as schizophrenia, depression, hyperactivity and attention symptoms [8]. DHA is essential for the growth and functional development of the brain in infants [9], and is also required for maintenance of normal brain function in adults [10, 11]. The FDA states it is safe to take up to 3 g of omega-3 per day to lower the risk for coronary heart disease (CHD) and maintain health [12]. Besides, the American Heart Association (AHA) recommends everyone eat fish (particularly fatty fish) at least twice a week. As omega-3 fatty acids are deemed important from authoritative bodies, supplementation in addition to food sources may need to be considered to help U.S. adults meet recommendations [13].

In the past 10 years, many people have taken omega-3 fish oil supplements for their health benefits. Hong Kong is one of the strongest markets for health supplements in Asia [14], and more and more fish oil products are being sold in the markets. These fish oils come from different sources, and product prices vary significantly. However, these products are labelled with same ingredient levels, namely EPA 180 mg/g and DHA 120 mg/g (Table 1). Confronted with this variety, consumers are eager to know whether these fish oils contain same EPA and DHA contents? Whether there is relationship between the EPA/DHA contents and the price or source? Are the more expensive fish oil products really of better quality? To answer these questions, a comparative analysis of the contents of EPA and DHA in commercially available fish oil is urgently needed.

**Table 1:** **The original sources, prices and contents of EPA and DHA in ten batches of fish oil capsule samples bought in Hong Kong (** ***n*** **= 3)**

Sample no.	Labelled origin	Price (HK$/g)	Labelled contents (mg/g)		Determined contents (mg/g)	
			EPA	DHA	EPA	DHA
1^a^	USA	1.98			200.45	201.45
2	Australia	1.68			148.05	137.15
3	USA	1.53			509.16	501.18
4	USA	1.49			418.54	645.70
5	Australia	1.02	180	120	132.44	123.26
6	USA	0.98			361.36	419.73
7	New Zealand	0.65			208.42	204.72
8	USA	0.64			166.42	125.33
9	USA	0.34			39.52	35.14
10	USA	0.23			112.19	153.11

^a^Samples are listed in order of decreasing price; that is, the most expensive are listed first.

The determination of EPA and DHA in fish oil has been reported by high-performance liquid chromatography (HPLC) [15, 16] and liquid chromatography-mass spectrometry (LC-MS) [17, 18]. However, the HPLC is not sensitive enough and the cost of LC-MS is high. In the present study, a new gas chromatography–mass spectrometry (GC-MS) method was developed for the analysis of fish oil. The samples bought from different drugstores in Hong Kong were analyzed to determine DHA and EPA contents. The results demonstrated that our method is highly precise and accurate, and is therefore suitable for the determination of EPA and DHA in fish oil. Significant variation in the contents of EPA and DHA in fish oil samples was founded, and there is not a linear relationship between price and contents of EPA and DHA. Higher price can not guarantee higher contents of EPA and DHA. But because fish oil is expensive and its health claims are so significant, this is one product the government should seriously monitor.

## Experimental

### Materials

The sources of the fish oil capsule samples are listed in Table 1. Corresponding voucher specimens were deposited in the School of Chinese Medicine, Hong Kong Baptist University.

### Reagents and chemicals

The standard compounds of eicosapentaenoic acid methyl ester, docosahexaenoic acid methyl ester and Supelco® 37 component fame mix were purchased from Sigma-Aldrich (St. Louis, MO, USA). The purity of these chemical standards was more than 98% by GC-MS.

*N*-hexane was used as a solvent in GC-MS analysis, which was purchased from the RCI Lab-Scan Limited (Bangkok, Thailand). Potassium hydroxide and sodium chloride of analytical grade was purchased from Uni-Chem (Shanghai, China). Boron trifluoride methanol complex solution (13-15% BF_3_ basis), used to carry out methyl esterification, was purchased from Sigma-Aldrich (St. Louis, MO, USA). Water was purified using a Milli-Q water system (Millipore; Bedford, MA, USA).

### GC–MS instrumentation and conditions

Shimadzu QP2010 GC-MS system (Kyoto, Japan) was used for qualitative and quantitative analysis of fish oil. DB-5 ms high resolution capillary column (Dikma Technologies. thickness: 0.25 μm, length: 30 m, diameter: 0.25 mm) was used for sample separation.

For temperature programming, the oven was maintained at 80°C for one minute and then increased at a rate of 10°C per minute to 250°C, the rate was then slowed to 8°C per minute until 280°C was reached and maintained for 5 min. Split injection was conducted with a split ratio of 10:1, and helium was used as the carrier gas at a rate of 0.8 ml/min, with the volume of injection as 1 μL. The mass spectrometer was operated in electron-impact (EI) mode. Pre-column pressure: 70 kPa. Injection temperature: 250°C. Ion source: EI (200°C). Interface temperature: 280°C. Electron energy: 70 eV. Solvent delay: 5.5 min. For qualitative analysis, the full scan mode was used and the scan range was 40–400 m/z. For quantitative analysis, selective ion mode was used, and m/z 79 was chosen as the ion fragment of EPA and DHA.

### Preparation of standard and sample solutions

The stock solutions of EPA methyl ester (5 mg/L) and DHA methyl ester (2.5 mg/L) were prepared in *n*-hexane and stored in the refrigerator. The working solutions were prepared by appropriate dilution of the stock solutions with *n*-hexane, and the resulting concentrations of were 1, 2.5, 5, 10, 20, 25 and 30 mg/L. DHA was prepared in serial dilutions of 2, 5, 10, 20, 40, 50 and 60 mg/L. Calibration standard solution (1 μL) was injected for GC-MS analysis.

The preparation of sample solutions was performed as previously described with modifications [19]. Samples were obtained from fish oil capsules by puncturing the capsule with a needle syringe. Each sample of approximately 60 mg was weighed accurately and placed in a centrifuge tube with a ground stopper. 3 mL potassium hydroxide methanol solution (0.5 M) was added. The contents were thoroughly mixed, and then the tube was filled with nitrogen gas, heated in a water bath at 60°C, shaken three times in the course of 20 min. When the oil droplets had disappeared completely and the solution was transparent, 3 mL boron trifluoride methanol complex solution was added, and the mixture cooled. Each tube was then filled with nitrogen gas, and placed in a water bath at 60°C for 5 min. Saturated sodium chloride solution of 2 mL and *n*-hexane of 2 mL were added and mixed well. After centrifugation (4000 rpm) for 10 min, the supernatant was drawn off, to be used as sample solution. Dilution of the supernatant is necessary in case its concentration falls out of the linear range. An aliquot of 1 μL supernatant was injected for GC-MS analysis.

### Assay validation and sample determination

Linearity for standards was determined with five data points over the concentration range of the working solutions. Precision was evaluated by six injections of the sample solution (batch 1) within one day. Repeatability was evaluated in intra- and inter-day assays of the fish oil sample FO1. The stability test was performed by analyzing the sample solution (batch 1) over a period of 24 h. The relative standard deviation (RSD) was taken as the measures of precision, repeatability and stability. Recovery of all the quantified constituents was determined by sample in different concentration levels using a mixture of standards with 50, 100 and 200% of the quantified levels of constituents in the samples. All fish oil samples were analyzed using this method, and the acid/ester conversion factor was set to 0.96.

## Results and discussion

### Optimization of hydrolysis and esterification conditions

EPA and DHA are present in fish oil in the form of various triglycerides. To generate a volatile DHA methyl ester for GC-MS analysis, EPA and DHA are released from the triglycerides by hydrolysis, and then methyl esterified. Various hydrolysis and methyl esterification conditions (different time, temperature, usage of nitrogen gas) were evaluated to obtain maximum extraction efficiency. The results demonstrated that incubation of fish oil (c.a. 60 mg) in potassium hydroxide methanol solution (0.5 M, 3 mL) at 60°C for 20 min achieved complete hydrolysis. Esterification with 3 mL boron trifluoride methanol solution at 60°C for 5 min provides the most methyl esters. Moreover, nitrogen used as protection gas ensures less oxidation and higher stability of the constituent contents. After further optimization, the best experimental conditions are shown in “*Preparation of standard and sample solutions*”.

### Chromatographic conditions and GC-MS identification

**Figure 2:**
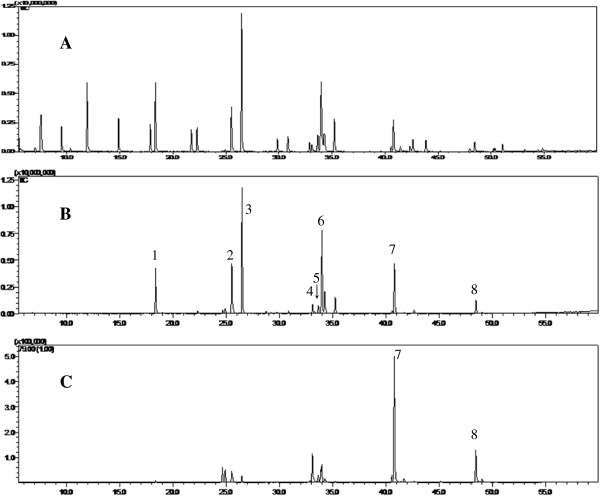
**Typical GC-MS chromatograms of (A) the mix standard and (B) fish oil sample at full scan mode; and (C) fish oil sample at selective ion mode.** 1, Methyl myristate (18.4 min); 2, Methyl palmitoleinate (25.5 min); 3, Methyl palmitate (26.5 min); 4, Methyl heptadecanoate (33.1 min); 5, Linoleic acid methyl ester (33.7 min); 6, Octadecenoic acid methyl ester (34.0 min); 7, Eicosapentaenoic acid methyl ester (40.8 min); 8, Docosahexaenoic acid methyl ester (48.5 min).

The chromatographic conditions such as temperature gradient and carrier gas flow rate were optimized to achieve satisfactory separation and sharp peak shape in the chromatograms. Full scan mode was used in qualitative tests because it provides more peaks for identification. For the qualitative analysis, apart from EPA (7) and DHA (8), 6 characteristic peaks of constituent chemicals had been successfully identified with the aid of the reference standard. They are methyl myristate (1), methyl palmitoleinate (2), methyl palmitate (3), methyl heptadecanoate (4), linoleic acid methyl ester (5), and octadecenoic acid methyl ester (6). The typical GC-MS chromatograms are shown in Figure 2A and 2B.

In quantitative test, selective ion mode (SIM) was used due to its higher sensitivity. Considering the abundance of fragment ions of EPA and DHA in mass spectra (Additional file 1: Figure S1), m/z 79 was used for calculating amount of EPA and DHA. The typical chromatogram is shown in Figure 2C.

### Validation of the analysis method

Results for assessment of the validity of the method are summarized in Table 2, 3 and 4. The data indicate good linearity between concentrations and peak areas of the analytes within the test ranges. The limits of detection (LOD) for EPA and DHA were found to be 0.08 ng and 0.21 ng, respectively. Therefore, the system was considered to be sensitive. The relative standard deviation (RSD) values of intra-day and inter-day variations were not more than 0.59% and 1.00% for EPA, and not more than 1.08% and 1.05% for DHA, respectively. The established method also had acceptable accuracy with average recovery of 100.50% and 103.83% for EPA and DHA. All these results demonstrate that the developed GC-MS method was sufficiently reliable and accurate and is therefore suitable for quantification of EPA and DHA in fish oil.

**Table 2:** **Linearity calibration curve factors, LOD and LOQ of EPA and DHA**

Analyte	Equation	Range (mg/L)	***R*** ^***2***^	LOD (ng)	LOQ (ng)
EPA	y = 41328.56x - 7.85	1-30	0.9999	0.08	0.15
DHA	y = 11863.62x + 4.62	2-60	0.9999	0.21	0.60

**Table 3:** **Precision, repeatability and stability of EPA and DHA**

Analyte	Precision (RSD, %, ***n*** = 3)	Repeatability (RSD, %, ***n*** = 3)				Stability (RSD, %, ***n*** = 6)
		Day 1	Day 2	Day 3	Inter-day	
EPA	0.38	0.38	0.59	0.16	1.00	0.85
DHA	0.32	0.08	0.32	1.08	1.05	0.63

**Table 4:** **Recovery of EPA and DHA**

Analyte	50%		100%		200%		Average	
	Recovery (%)	RSD (%)	Recovery (%)	RSD (%)	Recovery (%)	RSD (%)	Recovery (%)	RSD (%)
EPA	104.10 ± 3.71^a^	3.56	99.85 ± 2.15	2.15	97.55 ± 0.80	0.82	100.50 ± 3.32	3.31
DHA	104.70 ± 1.94	1.85	101.22 ± 2.38	2.35	105.56 ± 2.42	2.29	103.83 ± 2.30	2.21

^a^Values shown are mean ± SD (*n* = 3).

### Sample analysis

**Figure 3:**
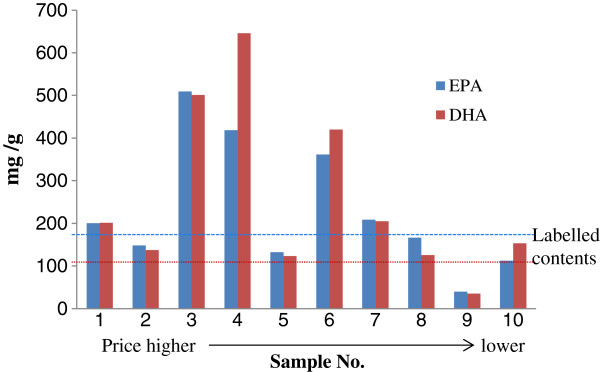
**The contents of EPA and DHA in ten batches of fish oil capsule samples as compared to price (** ***n*** **= 3).**

The present method was successfully applied to the quantification of EPA and DHA in fish oil capsule samples, and the results are summarized in Table 1 and Figure 3. The results reveal significant variation in the contents of the quantified components in fish oil samples. Such variations may be mainly due to the source and processing of the fish oil.

Fish oils are commercially produced from cold water fatty fish, including salmon, tuna, sardines, shellfish, and herring. The contents of EPA and DHA in theses fishes are significantly varied [20, 21]. During the filling of soft capsules, poor quality fish oil or even vegetable oil may be added thereby adulterating good quality fish oil [22]. Adulteration can induce unstable contents of EPA and DHA in the final products, which make the actual contents do not match the label.

Figure 3 also shows that the relationship between the price and the contents of DHA and EPA are insignificant. In other words, according to the prices for the various fish oil sold in markets, it was clear that the contents of EPA and DHA did not always correlate with price. For example, sample 3, 4 and 6 contained higher contents of EPA and DHA but it was cheaper than samples 1, 2 and 5. Distinctly, the classification of various prices of fish oil actually did not distinguish relative quality. This finding confirms the need for developing a reliable evaluation method to ensure the quality of fish oil products.

## Conclusions

A GC-MS method was developed for determination of EPA and DHA content in fish oil capsules. A comprehensive evaluation of the developed method was conducted, and the method was shown to be highly sensitive, reproducible and accurate. Samples of 10 commercial fish oil capsule samples bought in Hong Kong retail stores were tested. The results demonstrated significant variation in the contents of EPA and DHA in the samples, and there is not a linear relationship between price and contents of EPA and DHA. Strict supervision of the labelling of the fish oil capsules is urgently needed.

## Electronic supplementary material

Additional file 1: Figure S1: The mass spectra of **(a)** EPA and **(b)** DHA methyl ester. (DOCX 65 KB)

## Abbreviations

EPA: Eicosapentaenoic acid
DHA: Docosahexaenoic acid
GC-MS: Gas chromatography–mass spectrometry
LOD: Limit of detection
LOQ: Limit of quantitation.
