# Supplementary material for: Comparative analysis of EPA and DHA in fish oil nutritional capsules by GC-MS
Source: Lipids Health Dis. 2014 Dec 13;13:190. doi: 10.1186/1476-511X-13-190 (PMC4290138; doi:10.1186/1476-511X-13-190)
Supplement: Supplementary file 1 — Additional file 1: Figure S1: The mass spectra of (a) EPA and (b) DHA methyl ester. (DOCX 65 KB) [file 12944_2014_1186_MOESM1_ESM.docx]

**Figure S1** The mass spectra of (a) EPA and (b) DHA methyl ester.
